# City-Specific Spatiotemporal Infant and Neonatal Mortality Clusters: Links with Socioeconomic and Air Pollution Spatial Patterns in France

**DOI:** 10.3390/ijerph13060624

**Published:** 2016-06-22

**Authors:** Cindy M. Padilla, Wahida Kihal-Talantikit, Verónica M. Vieira, Séverine Deguen

**Affiliations:** 1 Department of Quantitative Methods in Public Health, EHESP School of Public Health-Sorbonne-Paris Cité, Rennes 35043, France; 2 IRSET—Research Institute of Environmental and Occupational Health, Rennes 35000, France; 3 Department of Environmental and Occupational Health, EHESP School of Public Health, Rennes, Sorbonne-Paris Cité 35043, France; wahida.kihal@ehesp.fr (W.K.-T.); severine.deguen@ehesp.fr (S.D.); 4 Program in Public Health, Chao Family Cancer Center, University of Irvine, Irvine, CA 92697, USA; vvieira@uci.edu

**Keywords:** infant mortality, neonatal mortality, cluster analysis, environmental nuisances, neighborhood deprivation, spatial modeling

## Abstract

Infant and neonatal mortality indicators are known to vary geographically, possibly as a result of socioeconomic and environmental inequalities. To better understand how these factors contribute to spatial and temporal patterns, we conducted a French ecological study comparing two time periods between 2002 and 2009 for three (purposefully distinct) Metropolitan Areas (MAs) and the city of Paris, using the French census block of parental residence as the geographic unit of analysis. We identified areas of excess risk and assessed the role of neighborhood deprivation and average nitrogen dioxide concentrations using generalized additive models to generate maps smoothed on longitude and latitude. Comparison of the two time periods indicated that statistically significant areas of elevated infant and neonatal mortality shifted northwards for the city of Paris, are present only in the earlier time period for Lille MA, only in the later time period for Lyon MA, and decrease over time for Marseille MA. These city-specific geographic patterns in neonatal and infant mortality are largely explained by socioeconomic and environmental inequalities. Spatial analysis can be a useful tool for understanding how risk factors contribute to disparities in health outcomes ranging from infant mortality to infectious disease—a leading cause of infant mortality.

## 1. Introduction

The infant mortality rate (infant deaths before 1 year of age per 1000 live births) and the neonatal mortality rate (infant deaths before 1 month of age per 1000 live births) are highly sensitive measures that reflect the economic development, general living conditions, social well-being and rates of illness of whole populations [1]. Major adverse birth outcomes such as preterm birth (<37 weeks gestation), low birth weight (<2500 grams) and small gestational age are leading causes of mortality and morbidity in infants worldwide [2,3,4,5].

In France, the infant mortality rate was halved between 1986 and 2003, falling from eight deaths per 1000 births to four, yet the rate appears to have plateaued, with rates of 3.6 deaths per 1000 births from 2005 to 2009 and 3.4 deaths per 1000 live births from 2010 to 2013. Due to the fact that in France the rate has reduced and then plateaued, the French infant mortality rate over the past decade contrasts with the progress made in many other European countries [6]. Furthermore, the French rate from 2010 to 2013 remains higher than that of many other European countries, seven of which (Finland, Luxembourg, Czech Republic, Slovenia, Sweden, Iceland, and Norway) record infant mortality rates of fewer than three deaths per 1000 live births [6].

While the major risk factors of neonatal mortality are endogenous (maternal health, healthcare and health system factors) in the post-neonatal period, there are also exogenous factors including accidents, poor nutrition and infectious disease [7]. In addition, infant and neonatal mortality are unevenly distributed by geographic region and social inequality [7]. Contextual factors, such as social and environmental exposures, which are known to be clustered in space, are well-documented as associated with adverse birth outcomes. Studies have shown that both infant mortality and its risk factors are more common among women of low socioeconomic status measured by occupation [8], unemployment [9], education [8], or neighborhood deprivation level [10]. Studies investigating the relationship between race/ethnicity and the spatial distribution of adverse birth outcome risk in Michigan (USA) have reported higher rates in racially segregated areas [11,12].

Other studies have demonstrated the association between environmental exposure (particularly air pollution) and adverse birth outcomes [13,14,15,16,17]. Few studies examine environmental exposures as determinants which could partially explain the spatial distribution of social health inequalities related to adverse birth outcomes [18,19,20,21].

The role of location in shaping the unequal landscapes of adverse birth outcomes today is still not completely clear. It is therefore important to identify and monitor the spatial patterns of adverse birth outcomes, so that timely area-oriented health interventions can be delivered based on local contexts and observable local environmental risks. Articles reporting on which disparities have changed over time have received little attention [22,23,24,25,26]. A Swedish cohort study suggests that social inequalities in infant and post-neonatal mortality have persisted over many decades [26]. To our knowledge, no previous temporal studies of infant or neonatal mortality have also considered location as a determinant of disparities, especially in the context of assessing the contribution of environmental factors to socio-spatial health inequalities.

The objective of this study is to identify—within the three large French MAs and the city of Paris—whether urban neighborhoods are characterized by an uneven distribution of infant and neonatal mortality rates, according to ambient air concentrations of nitrogen dioxide (NO_2_) and the level of socioeconomic deprivation between two distinct periods of time.

## 2. Methods

### 2.1. Study Setting and Population

#### 2.1.1. MAs

**Figure 1:**
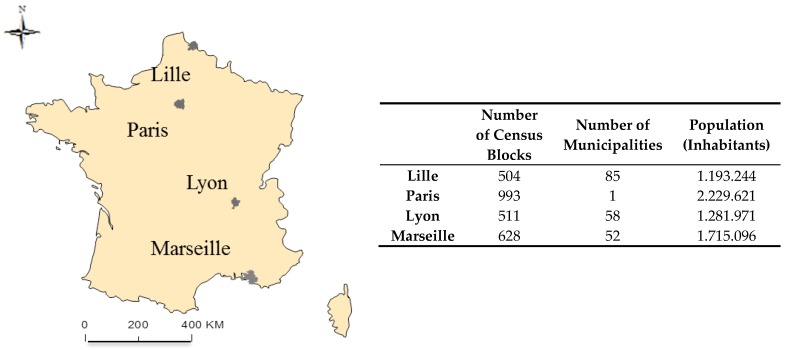
Location of the three metropolitan areas and the city of Paris in France.

The present research is part of the ongoing Equit’Area project. We achieved our research objectives through an ecological study conducted in three French MAs: Lille, Lyon and Marseille, as well as in Paris (Figure 1). These areas are of particular interest because their urban landscapes are contrasted in terms of certain significant demographic and socioeconomic characteristics. A detailed description of the socioeconomic and demographic variables and NO_2_ concentrations for the two time periods and four geographic areas are provided elsewhere [27]. The statistical unit is the IRIS (Ilots Regroupés pour l’Information Statistique)—a sub-municipal French census block defined by the National Institute of Statistics and Economic Studies (INSEE) [28]. This is the smallest administrative unit in which socioeconomic and demographic data are available in France. This geographical unit averages 2000 inhabitants and is constructed to be as homogenous as possible in terms of socio-demographic characteristics and land use. The IRIS is similar to the U.S. Census Block Group unit, for which socioeconomic data are available on population sizes ranging from 600 to 2000 residents [29]. The delineations of the census blocks provided by INSEE also take into account the urban landscape and obstacles that could divide it, such as major traffic roads, green spaces and water bodies.

Based on the data reflecting a decline followed by a plateau in infant mortality, the study period was stratified by two periods of 4 years (2002–2005 and 2006–2009) within the three large French MAs and the city of Paris.

#### 2.1.2. Study Population

Infant and neonatal mortality rates were computed as the ratio of total number of cases to total number of births summed over the two time periods, 2002–2005 and 2006–2009, for each census block in the three MAs and for the city of Paris. Infant death data were collected from death certificates recorded in the City Halls of each metropolitan area. All cases were geocoded to the census block of the birth address. We obtained permission from the French National Commission for Digitalized Information and Liberty (CNIL; 911149) to geocode and analyze the health data. Birth addresses were matched to the corresponding census blocks using map databases (Correspondence Address-Urban Areas, 2004)—software issued by INSEE. The annual number of births by census block is available from the INSEE institute.

Census blocks without any births (for example, those containing only industrial buildings, or a park) were excluded from the analysis. We also excluded 24 census blocks (4.5%) in Lille, 12 census blocks in Lyon (2.3%) and 50 census blocks in Marseille (7.9%) for which no NO_2_ measures were available, which represent 2.7%, 2.8% and 4.3% of deaths excluded respectively. None of the census blocks in the city of Paris were excluded. The final dataset included 472, 492, 565 and 935 census blocks for Lille, Lyon, Marseille MAs and the city of Paris, respectively. The same number of census blocks was analyzed for both time periods.

### 2.2. Potential Risk Factors

#### 2.2.1. Deprivation Index

The potential social spatial risk factor of interest is a contextual deprivation index to characterize socioeconomic neighborhood level. We constructed a composite neighborhood deprivation index at census block level that measures area level deprivation. Socioeconomic and demographic data estimated from the 1999 (for the first period 2002–2005) and 2006 (for the second period 2006–2009) national censuses were selected to construct the deprivation index for each period and each metropolitan area separately. The domains covered by the index are: family, immigration status and mobility, employment and income, and education and housing. Successive principal-component analyses were used to synthesize this information. The inertia of the first component was maximized by deleting all variables only weakly correlated with this first component and variables with a below-average contribution (more details available in the article by Lalloué *et al.* [30]). Previous ecological studies have demonstrated this index’s ability to capture environment-related socio-spatial inequalities in France [19,20,27]. The measure of neighborhood deprivation was categorized into three groups (low, moderate and high deprivation) according to the index distribution tertiles.

#### 2.2.2. Traffic Exposure Assessment

The potential environmental spatial risk factor of interest is the average annual nitrogen dioxide concentration (NO_2_, µg/m^3^) for each census block. Annual average ambient concentrations of nitrogen dioxide (NO_2_) were modeled by the four local air quality monitoring networks corresponding to the four areas of the study, for each census block and throughout the two time periods. The four networks used different deterministic models: Atmospheric Dispersion Modeling System (ADMS) Urban for the Lille MA [31,32], SIRANE for the Lyon MA [33,34], ESMERALDA for the Paris MA [32] and STREET for the Marseille MA [32]. They integrated meteorological data (air temperature, wind speed and direction, relative humidity, and barometric pressure, all supplied by the French meteorological service), emission sources (according to their contribution to ambient air pollution) and background pollution measurements as input parameters. Selected emission sources were linear (main roads), surface (diffuse road sources and residential and tertiary emissions) and important point sources. They develop a homogeneous methodological approach to describe and characterize disparities in nitrogen dioxide exposure at census block scale. NO_2_ is a good tracer of pollution generated by traffic and other combustion sources. Previous studies have demonstrated that exposure to dioxide may vary substantially among socioeconomic groups [35,36,37], be related to adverse birth outcomes [38,39,40,41,42] and may also have toxic effects directly related to the fetus [43].

### 2.3. Statistical Methods

#### 2.3.1. Spatial Analysis

This study requires a rigorous methodology in order to minimize ecological biases and account for the dependency of spatial units. To represent the geographic variation of the relative risk of infant or neonatal mortality, the classic approach is to calculate the standardized mortality ratio (*i.e.*, the ratio between the observed number of cases and the expected number of cases). Nevertheless, the standardized mortality ratio could be problematic in case of rare events, which can produce estimations with very large variance that are not representative of the truth. The Generalized Additive Model (GAM) has been applied to take into account spatial autocorrelation and heterogeneity in the associations of interest. This model takes into account the spatial dependence of the data and infant mortality rate variability that is due to the small number of events per geographic unit, by using a locally weighted regression smoother (LOESS) to account for geographic location as a possible predictor of infant mortality rate [44,45,46].

#### 2.3.2. Global and Local Spatial Analysis

We estimated census block level infant and neonatal mortality risk using GAMs [47,48,49,50], a form of non-parametric or semi-parametric regression having the ability to analyze contextual data while adjusting for covariates. We modeled location, a potential proxy measure of unknown exposure or uncontrolled risk factors, using a smooth (S) of longitude (X) and latitude (Y) with a Poisson link function:

Log [p (X, Y)] = S (X, Y) + offset (birth) + γ’Z
(1)

where the left-hand side is the logarithm of the infant or neonatal mortality risk at the IRIS census block’s centroid (X, Y), according to the size of the population (offset (birth)), and γ is a vector of parameters associated with Z, the vector of covariates. The Poisson function was used in the model because the outcomes are countable data within a census block. The model is semi-parametric because it includes components that are both non-parametric (the smooth function for location) and parametric (the covariates). Without the smooth function, S (X, Y), the model becomes an ordinary Poisson regression on the covariates. Omitting the covariates produces a crude (unadjusted) map. We used the GAM package within the R statistical software (which is an implementation of the GAM framework of Hastie and Tibshirani [50]) to perform the generalized additive modeling, and ArcView 9.3 software (ESRI, Inc., Redlands, CA, USA) to map the results of our analyses.

For our analyses, we implemented a LOESS smooth which adapts to changes in data density previously used in case-control studies [44,51,52,53] as well as in a single ecological study [19]. The amount of smoothing depends on the percentage of the data points in the smoothing window, referred to as span size. We determined the optimal amount of smoothing for each map by minimizing the Akaike’s Information Criterion (AIC). Small span sizes produce precise surfaces and larger span sizes produce smoother surfaces. As the span size increases, the amount of bias in the model fit increases, and variance decreases. GAMs also provide a framework for testing hypotheses. There are a number of ways to test the global null hypothesis that disease status does not depend on location, *i.e.*, that the spatial distribution of the map is homogeneous. Similar to variance analysis in ordinary linear regression, we examined the overall significance of location using the difference of the deviances of the complete model (Equation (1)) and the reduced model omitting the smoothing term. The R software provides an approximate *p*-value for this statistic assuming a chi square distribution. Because the latter assumption is in general not true for GAMs, we calculated the *p*-value using a permutation test [54]. To test the null hypothesis of no association between infant and neonatal mortality rate and location, we randomly reassigned the coordinates of the census blocks while keeping the case counts, population, and covariates fixed. From the null permutation we sampled distribution 999 times in addition to the original model. For each permutation, we ran the GAM using the optimal span of the original data and computed the deviance statistic. We divided the rank of the observed value by 1000 to obtain the permutation *p*-value. If the deviance global statistic indicated that location was significant at the 0.05 level, we then identified areas with significantly increased or decreased risk. We did this by obtaining a distribution of the log risk at every census block using the same set of permutations we used for calculating the global statistics. We identified areas of significantly elevated risk (“hot spots”) as all census blocks ranking in the upper 2.5% of the census block distributions, and denoted these areas with a black contour line in the resulting maps [52,55].

We first performed spatial analyses for infant and neonatal mortality at two distinct time periods, using the crude model to determine the unadjusted geographic variation. Spatial patterns in the underlying crude analysis could be due to a number of factors having a geographic component. In this study, we were primarily interested in spatial patterns capable of being explained by the deprivation index or the NO_2_ concentrations. To assess the contribution made by these factors to the underlying spatial patterns, we performed adjusted analyses using the deprivation index alone, NO_2_ concentrations alone, the deprivation index and NO_2_ concentrations together, and with their interaction. Comparing the two periods, we sought to answer two main study questions: (1) Do significant areas of increased infant or neonatal mortality appear in the first, second or both periods, and, if so, is the location the same between the two periods? (2) After adjustment for risk factors (socioeconomic deprivation, NO_2_ concentrations or both), do the spatial patterns persist?

## 3. Results

### 3.1. Social Heath Inequality Trends

In our study, between the two time periods, infant mortality rates moved from 3.53 to 3.02 per 1000 live births in Marseille MA (*p* = 0.64) and from 3.49 to 3.19 per 1000 live births in the city of Paris. We found a shift in neonatal mortality rates, from 2.08 to 1.91 per 1000 live births in Marseille MA (*p* = 0.90) and from 2.53 to 2.37 per 1000 live births in the city of Paris (*p* = 0.21). In Lyon MA, the neonatal mortality rate remained fairly stable, at 2.77 to 2.72 per 1000 live births (*p* = 0.68) and from 3.88 to 3.95 per 1000 live births for the infant mortality rate (*p* = 0.32). Conversely, the Lille MA indicates a significant decrease in both infant mortality rates: from 4.55 to 3.69 per 1000 live births (*p* = 0.04) and neonatal mortality rates: from 3.23 to 2.27 per 1000 live births (*p* = 0.005). Descriptive statistics related to NO_2_ concentrations, the deprivation index and the relationships between each other are described in detail by Padilla *et al.* [27].

Table 1 presents the infant and neonatal mortality rates per 1000 live births over the two time periods (Period 1: 2002–2005, Period 2: 2006–2009) for the three MAs and the city of Paris, across all categories of the deprivation index. Despite overall reductions of the rates in the Lille MA, Marseille MA and the city of Paris, we found large socioeconomic disparities in infant and neonatal mortality. In the least deprived census blocks, the rates of infant and neonatal mortality increased in the Lille MA, Lyon MA, and Paris. In the most deprived census blocks, the rates of infant mortality decreased in the Lille MA, Marseille MA, and Paris, and for neonatal mortality, the rates decreased in the Lille MA and Paris.

Table 2 compares infant and neonatal mortality rates per 1000 live births stratified by NO_2_ and deprivation index for two time periods in the 3 MAs and Paris. In the Lyon MA census blocks that are most deprived and have the highest NO_2_ levels, the rate of infant mortality decreased (Table 2A). Conversely, the rate of infant mortality increased in the most deprived areas with the lowest NO_2_ levels (Table 2B). The rate of neonatal mortality also decreased between the time periods in areas with the lowest deprivation and lowest NO_2_ levels (Table 2B). In those areas of Paris that are most deprived with the highest NO_2_ levels (Table 2A), the rates of infant and neonatal mortality decreased - yet rates increased in the most deprived areas having the lowest NO_2_ levels (Table 2B).

### 3.2. Patterns of Spatial Variation in Health Risk

**Figure 2:**
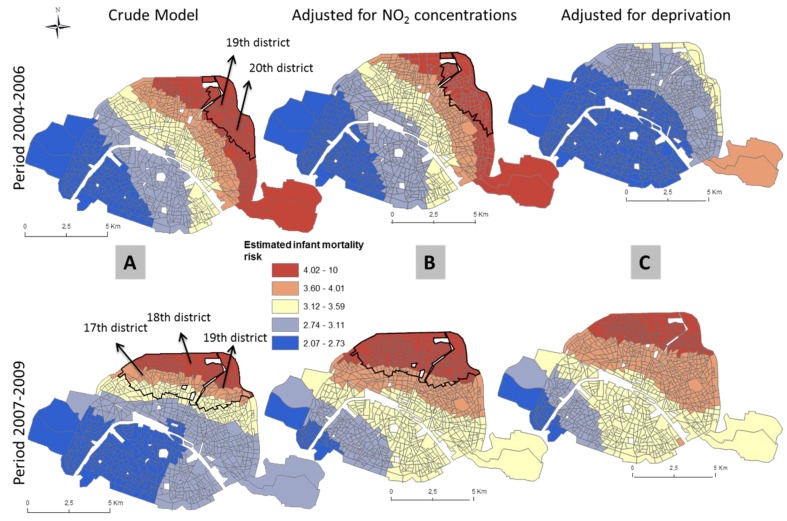
Prevalence of infant mortality in Paris at two time periods estimated using GAMs for the crude analysis (**A**); and adjusted for NO_2_ concentrations (**B**) and deprivation index (**C**). Solid lines identify areas with statistically significant elevated risk (hotspots).

**Figure 3:**
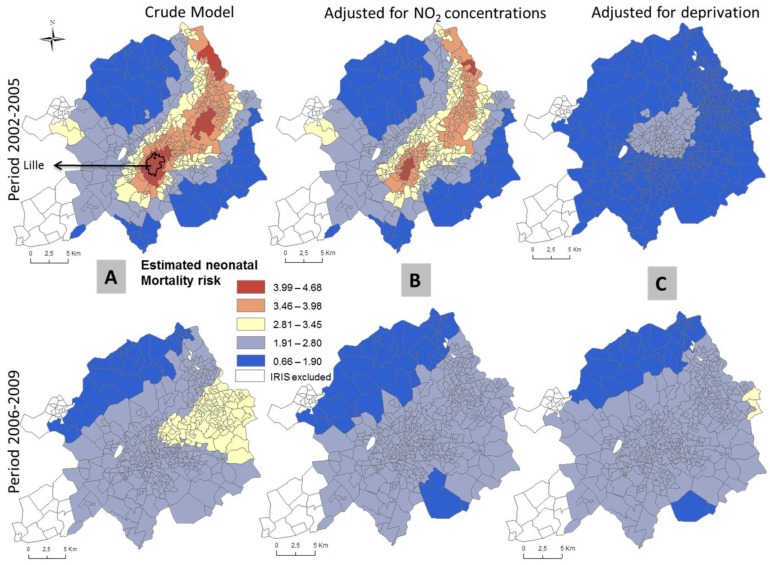
Prevalence of neonatal mortality in the Lille MA at two time periods estimated using GAMs for the crude analysis (**A**); and adjusted for NO_2_ concentrations (**B**) and deprivation index (**C**). Solid lines identify areas with statistically significant elevated risk (hotspots).

**Figure 4:**
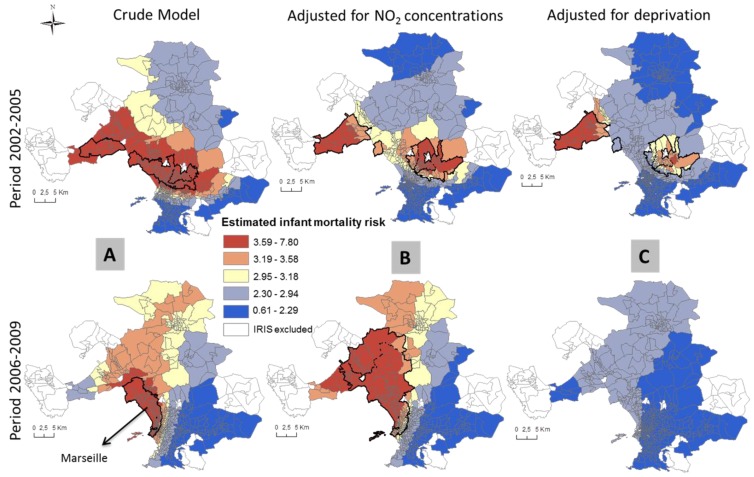
Prevalence of infant mortality in the Marseille MA at the two time periods estimated using GAMs for the crude analysis (**A**); and adjusted for NO_2_ concentrations (**B**) and deprivation index (**C**). Solid lines identify areas with statistically significant elevated risk (hotspots).

**Figure 5:**
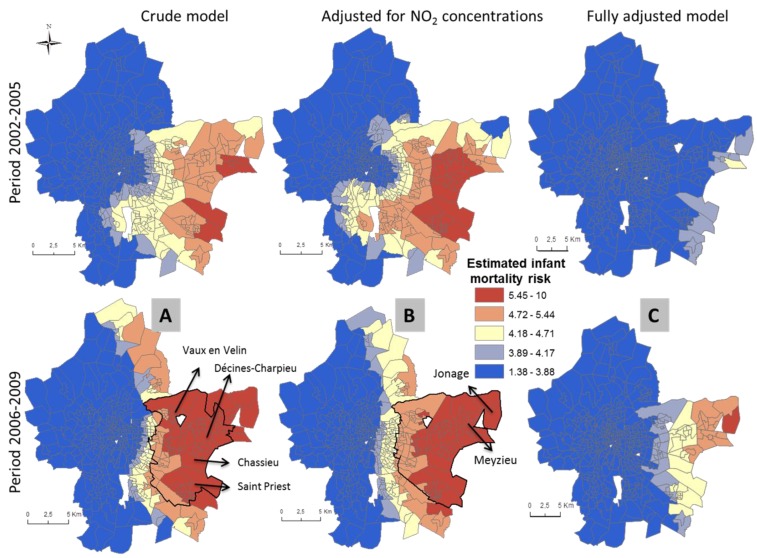
Prevalence of infant mortality in the Lyon MA at two time periods estimated using GAMs for the crude analysis (**A**); adjusted for NO_2_ concentrations (**B**) and fully adjusted for NO_2_ concentrations and deprivation index (**C**). Solid lines identify areas with statistically significant elevated risk (hotspots).

In unadjusted models, areas of statistical significance were identified in the 3 MAs and Paris (Table 3 and Table 4). In Paris, spatial patterns differ between the two time periods. Statistically significant areas of increased unadjusted infant mortality are located in the 19th and 20th districts in the first period and the 17th, 18th and 19th districts in the second time period (Figure 2). In the Lille (Figure 3) and Marseille (Figure 4) MAs, the number of significant areas differs between the two time periods; some areas of statistical significance for increased unadjusted neonatal and infant mortality during the first time period were no longer present in the second time period (Table 3 and Table 4). Conversely, in Lyon MA, significant areas of increased unadjusted infant and neonatal mortality located in the towns of Vaux en Velin, Decines and Saint Priest, to the east of the map appear only in the second time period (Table 3 and Table 4, Figure 5). There is a visible spatial gradient of increased unadjusted infant mortality risk from southwest to northeast Paris (Figure 2) and from northwest to southeast Lyon MA (Figure 5).

In the Lille MA and the city of Paris, for both time periods, and after adjusting for deprivation alone, there are no longer any areas of statistically significant increased risk of infant mortality, and global *p*-values become non-significant (Table 3, Figure 2). This suggests that the spatial variability of infant mortality risk was largely due to the geographic distribution of the deprivation index. In the Marseille MA, adjusting for deprivation or NO_2_ concentrations does not completely explain the spatial variability of infant mortality risk in the first time period; the global *p*-value remains significant—*p* = 0.004 and *p* = 0.006, respectively—whereas in the second time period, deprivation is an important spatial predictor (global *p*-value = 0.103; Table 3, Figure 4). For all the MAs and the city of Paris, NO_2_ concentrations do not explain the infant mortality spatial patterns. After adjustment for NO_2_ concentrations alone, the same areas remain statistically significant (for Lyon, global *p*-value = 0.004). Conversely, the spatial variability of neonatal mortality in the Lille MA is largely due to NO_2_ concentrations (global *p*-value = 0.173; Table 4). After adjustment, Figure 3 shows that areas of statistically significant increased neonatal risk in the unadjusted analysis for the Lille MA are no longer significant, although risks in those areas are still on the rise. In Lyon, NO_2_ concentrations explain some spatial variability, yet may not completely explain the relationship between neonatal mortality and location (global *p*-value = 0.06; Table 4). For both MAs, spatial patterns are influenced by the two risk factors, with a higher *p*-value and reduced hot spot in the fully-adjusted models.

## 4. Discussion

In this study, we explore the contribution made by NO_2_ to the spatial distribution of social health inequalities related to infant and neonatal mortality using GAMs. Our results highlight city-specific patterns of spatial inequalities in infant and neonatal mortality. We observed that infant and neonatal mortality was consistently highest among the most deprived census blocks, regardless of location or time period. However, patterns of change in mortality over time for the three MAs and the city of Paris differed by deprivation level. Specifically, the Lille MA and the city of Paris have reduced their social health inequalities between the two time periods, whereas socioeconomic inequalities persisted in terms of neonatal mortality in the Lyon and Marseille MAs. We demonstrated that during the second period, areas of increased infant and neonatal risk shifted geographically within the city of Paris; became apparent in the Lyon MA; and disappeared or decreased for the Lille and Marseille MAs. The influence of socioeconomic and environmental factors in the geographic variation of these diseases differs according to outcome, area and time period. To our knowledge, no studies have compared the effects of socioeconomic deprivation and NO_2_ concentrations on infant and neonatal mortality across two time periods.

### 4.1. Trends in Inequalities

Despite the impressive overall decrease in infant and neonatal mortality for the Lille and Marseille MAs and the city of Paris, large socioeconomic inequalities persisted in the Lyon and Marseille MAs between the two periods. In the Marseille MA, social inequalities in neonatal mortality are apparent, with an increase in mortality among the most deprived census blocks and a decrease among the least deprived census blocks. In the Lyon MA, infant and neonatal mortality increased across all census blocks between the two time periods, although the increase was higher in the most deprived census blocks. For the Lille MA and the city of Paris, mortality increased in the least deprived census blocks, but saw substantial reductions in the most deprived census blocks. Previous studies have demonstrated that the decline in infant and neonatal mortality are not equally distributed and that social inequalities persist [24,25,26,56]. They also demonstrated that time trends differed between neonatal and post-neonatal deaths [24,26,57]. Our results show that inequalities in infant and neonatal mortality are present throughout France. Although greater reductions in mortality over time have been observed in the most deprived census blocks, mortality is still higher in these areas in comparison with the least deprived census blocks. In our study, larger social inequalities were found for neonatal mortality; the major risk factors are endogenous (healthcare and health system characteristics), suggesting a continuing gap in access to high-quality health care across deprived groups. Some studies highlight the fact that maternal education also plays an important role in infant mortality rates over time [26].

Our results also show that NO_2_ concentrations contribute to differences in mortality rates in the Lyon MA and the city of Paris across deprivation levels. In the Lyon MA, neonatal mortality increased between time periods for both deprivation groups, but when we stratified by census block using the highest and lowest NO_2_ levels, neonatal mortality decreased for the least deprived census blocks and increased for the most deprived census blocks. Some researchers hypothesize that air pollution can create or accentuate existing social inequalities [58,59,60]. Populations having low socioeconomic status may be more frequently and more intensely exposed to pollution and/or are perhaps more susceptible to pollution than those with a higher socioeconomic status [27].

### 4.2. Spatial Inequalities

In the Lille MA and the city of Paris, socioeconomic deprivation explains a large portion of spatial variability in infant mortality during the two periods. After adjusting for deprivation alone, there are no longer any areas of statistically significant risk of infant mortality. Socioeconomic inequalities in birth outcomes are among the most robust findings in perinatal epidemiological research [61]. Studies have shown that both infant mortality and its risk factors are more common among women of low socioeconomic status as measured by occupation [8], unemployment [9], education [8], or neighborhood deprivation level [10]. 

Our results for infant mortality show that spatial patterns in the MAs and Paris are not completely explained by NO_2_ concentrations. Conversely, NO_2_ concentrations can explain a substantial part of the spatial variability of neonatal mortality in the Lille MA. After adjustment, statistically significant areas of neonatal risk are no longer apparent. Previous studies have demonstrated that air pollution could contribute to preterm births, intrauterine growth and perinatal mortality [62,63,64,65].

In the Lille and Lyon MAs, fully-adjusted analyses show that air pollution and deprivation explain most of the spatial pattern, resulting in areas that have a less pronounced neonatal mortality risk hotspot. This result is supported by previous work suggesting that air pollution may be related to social inequalities in health outcomes [16,66]. Lastly, environmental risk factors are often suspected of being at work in infant and neonatal mortality because of the excess risk in areas of high exposure—but our results suggest that air pollution exposure and socioeconomic inequalities are highly co-located.

### 4.3. Usefulness of Spatial Analysis in Public Health

Small area health studies that identify geographic areas having relatively high infant mortality risks, and that adjust for deprivation index and NO_2_ exposure, can provide important data to inform local health policies. This tool allows the early identification of areas in need of policymakers to focus the scope of prevention/intervention programs and tackle the social gradient in health by providing more effective—and more local—interventions the better to respond to individual needs, to achieve more efficient distribution of public resources. Policy makers could then focus on an appropriate direction, depending on the particular area’s need. This geospatial methodology allows scientists to identify those clusters in need of development, and thus to implement interventions and reorient health services.

### 4.4. Relevance of Infectious Disease to Spatial Analysis

Although our work focused on overall infant and neonatal mortality, similar analyses could be applied to infectious diseases such as diarrhea, toxoplasmosis, and rubella, which are leading causes of infant mortality. In the developed world, public health initiatives such as childhood vaccination have helped reduce the effect of infectious disease on both fetal and neonatal death, and long-term morbidity [67]. However, infectious disease still takes a major toll on pregnant women, their fetuses and children throughout the developing world. Indeed, it has been estimated that 30%–40% of neonatal deaths worldwide (totaling 4,035,000 in 2001 according to WHO) are associated with infectious disease [67]. Even in the developed world, there remain considerable challenges for the obstetrician and neonatologist in the management of infectious disease during pregnancy and in newborns [68].

The spatial and temporal distributions of infectious disease are important for public health policies in making relevant recommendations for the population living in high-risk areas [69]. Green *et al.* in Manitoba, Canada demonstrated the value of using a diverse set of spatial techniques to better understand the dynamics of an enteric disease such as campylobacter infection [70]. A systematic review related to dengue risk mapping concluded that although descriptive maps showing dengue case hotspots were useful for identifying high-risk areas, their applicability in public health contexts remains to be established [71]. Spatial analysis could be a useful tool for researchers studying transmission dynamics and pathogens’ circulation of infectious diseases and GAMs would allow visualization of those patterns while accounting for other risk factors. To that end, spatial models related to infectious disease need to consider serological profiles, circulating viral serotypes/genotypes and human movements [71] to improve our understanding of factors that could explain the spatial distribution [72,73].

Infectious disease risk maps can be powerful tools for the facilitation of decision-making in public health, ranging from surveillance to prediction maps. Previous studies have also been used to implement antimalarial interventions at household level [74]; identify clusters of hypoplastic left heart birth defects, and assess the genetic and environmental factors contributing to hypoplastic left heart defects [75]. The further development of this tool, useful for both research and public health applications, will depend on the acquisition and availability of data with high-quality spatial and temporal resolution and extensive covariates. Previous studies have demonstrated the public health relevance of predicting high-risk geographic areas of infectious disease such as visceral leishmaniasis [73] and dengue fever [71]. GAMs have been successfully applied to emergency department data, which is often used for surveillance of infectious disease [76].

### 4.5. Strengths and Limitations

We know that geographic level is an important consideration in such investigations, irrespective of the analytical method used. There is a small but growing number of studies utilizing zone design methodologies to evaluate the spatial patterns of health outcomes and local risk factors related to area level deprivation [77,78]. These studies demonstrate that size has to be as small as possible to maximize the homogeneity of specified variables within each area, as well as large differences between areas. The use of the smallest French census area level (2000 inhabitants with available data) in this study could be considered a strength in the spatial analysis of infant and neonatal mortality. Some authors suggest that analyses using small area census units can be improved through the use of advanced geographic methods, such as spatial smoothers and cluster detection methods [12,79].

One strength of our approach is that we modeled health risks using GAMs. Compared to classic cluster detection methods, GAMs include a non-parametric term to account for spatial variation in the health risk while simultaneously adjusting for potential confounders, and a framework for hypothesis testing using permutations to determine areas of significantly elevated risks where location is significant in the global tests. Unlike other well-known cluster methods, the GAM method is not limited by clusters of predefined shapes (*i.e.*, circle or ellipse). Moreover, we can identify areas of significant elevated risk (hotspots) by delineating areas that are in the upper 97.5% of the permutation distribution.

A further strength is the use of modeled air pollution concentrations at census block scale. This air modeling procedure provides unbiased estimates of exposure to ambient air pollution. In 2010, Jerrett *et al.* demonstrated the effectiveness and reliability of this type of model by using it in a study assessing air quality and health effects, rather than using surrogate air pollution measures (e.g., average pollutant concentrations at fixed ambient monitoring stations, distance to monitoring sites, vehicular traffic emissions, proximity to highway, and distance to main roads) [80].

Interpretation of our findings must also consider certain weaknesses- particularly in exposure assessment. Because individual exposure measurements were unavailable, we used NO_2_ mean concentration estimates at census block level. At this time, only NO_2_ concentrations were available from the official air quality monitoring associations, but further research should also consider other pollutants such as Particulate Matter (PM) and environmental hazards including industry and noise) to analyze the spatial distribution of birth outcomes. However, NO_2_ is a good tracer of pollution generated by traffic and other combustion sources, and presents a higher level of spatial heterogeneity than other pollutants [35].

In this study, we adjusted for deprivation index, but additional risk factors are hypothesized in the literature—for which we do not, unfortunately, have individual level data. Incorporating information from maternal interviews or detailed medical records, which we did not have available for this study, could help check for the potential influence of these factors. For example, birth weight, gestational age, the age of the mother, and the parity of the newborn have been linked with risk of infant mortality. Some maternal lifestyle behaviors [63,81,82] have also been linked, including the consumption of alcohol, smoking, using drugs, maternal nutritional deficits and access to health care [82,83,84,85].

## 5. Conclusions

In summary, we have identified city-specific patterns of spatial inequalities in infant and neonatal mortality over time. The influence of deprivation index and NO_2_ exposure in the geographic variation of these outcomes differs depending on the area and time period. Whereas socioeconomic status explains a large part of the spatiotemporal variability of infant mortality, NO_2_ concentrations only appear to explain some of the spatial variability of neonatal mortality in the Lille MA. The role of environmental exposures should be interpreted cautiously, since NO_2_ alone was taken into account. Nevertheless, the data suggest that environmental exposures may influence observed socioeconomic inequalities. Visualizing and exploring the spatial patterns of infant and neonatal mortality risk is important for generating mechanistic hypotheses, targeting high-risk neighborhoods for monitoring and implementing maternal and child health interventions and prevention programs, as well as for evaluating the need for health care services. Environmental surveillance and spatial statistical analyses should be conducted regularly by local health authorities to identify and monitor the impact of environmental and social changes on health in general and on birth outcomes in particular.

## Figures and Tables

**Table 1:** Infant and neonatal mortality rates per 1000 live births in the most and least deprived census blocks for two time periods (P1: 2002–2005; P2: 2006–2009) in the three French metropolitan areas and the city of Paris.

Study Areas	Infant Mortality Rates (‰)							Neonatal Mortality Rates (‰)					
	N	Most Deprived Census Blocks ^†^			Least Deprived Census Blocks ^‡^			Most Deprived Census Blocks			Least Deprived Census Blocks		
		P1 *	P2 *	Percent Change (%) **	P1	P2	Percent Change (%)	P1	P2	Percent Change (%)	P1	P2	Percent Change (%)
**Lille**	472	5.85	5.26	−10.10	2.65	2.93	+10.36	4.20	3.51	−16.53	1.75	2.30	+31.93
**Lyon**	492	4.54	5.38	+18.62	2.60	2.98	+14.15	3.04	3.72	+22.64	1.68	1.86	+10.47
**Marseille**	565	4.32	3.81	−11.72	2.29	1.98	−13.60	2.37	2.58	+9.01	1.57	1.05	−33.15
**Paris city**	935	4.12	3.99	−3.03	2.46	3.13	+27.45	2.96	2.76	−6.56	1.77	2.50	+41.36

^†^ The most deprived census blocks correspond to the third tertile of the deprivation index distribution; ^‡^ The least deprived census blocks correspond to the first tertile of the deprivation index distribution; * For the three metropolitan areas and Paris, ** Calculated as: ((mortality at P2‰ − mortality at P1‰)/mortality at P1‰) × 100. Example: −10.10% = ((5.26‰ − 5.85‰)/5.85‰) × 100.

**Table 2:** Infant and neonatal mortality rates per 1000 live births in the most and least deprived census blocks for two time periods (P1: 2002–2005; P2: 2006–2009) in the three French metropolitan areas and the city of Paris, stratified by census blocks with the highest (**A**) and lowest (**B**) NO_2_.

(A)													
Highest NO_2_ Census Blocks ^‡^		Infant Mortality Rate (‰)						Neonatal Mortality Rate (‰)					
		Most Deprived Census Blocks ^†^			Least Deprived Census Blocks ^†^			Most Deprived Census Blocks			Least Deprived Census Blocks		
	N	P1 *	P2 *	Percent Change (%) **	P1	P2	Percent Change (%)	P1	P2	Percent Change (%)	P1	P2	Percent Change (%)
**Lille**	472	5.91	5.49	−7.09	3.78	4.48	+18.34	4.58	3.82	−16.52	2.91	3.28	+12.82
**Lyon**	492	*4.33*	*3.97*	−*8.36*	2.36	3.71	+57.27	2.71	2.95	+9.03	*1.35*	*3.09*	+*129.3*
**Marseille**	565	3.72	4.54	+21.97	0.0	4.84	---	1.98	2.86	+44.11	0.0	3.87	---
**Paris city**	935	*5.56*	*4.70*	−*15.54*	2.52	2.91	+15.13	*4.24*	*3.30*	−*22.22*	1.56	2.30	+47.24
**(B)**													
**Lowest NO_2_ Census Blocks ^‡^**		**Infant Mortality Rate (‰)**						**Neonatal Mortality Rate (‰)**					
		**Most Deprived Census Blocks**			**Least Deprived Census Blocks ^†^**			**Most Deprived Census Blocks**			**Least Deprived Census Blocks**		
	**N**	P1 *	P2 *	Percent Change (%) **	**P1**	**P2**	**Percent Change (%)**	**P1**	**P2**	**Percent Change (%)**	**P1**	**P2**	**Percent Change (%)**
**Lille**	472	5.67	4.79	−15.50	2.19	2.46	+12.17	3.51	2.54	−27.73	1.27	1.92	+51.63
**Lyon**	492	*6.16*	*6.96*	+*12.8*	3.10	3.40	+9.90	4.75	4.92	+3.37	*2.18*	*1.80*	−*17.32*
**Marseille**	565	3.94	3.72	−5.73	2.00	2.17	+8.19	2.63	3.10	+17.83	1.24	0.9	−27.18
**Paris city**	935	*3.81*	*4.10*	+*7.68*	2.37	3.01	+27.21	*2.61*	*2.71*	+*4.03*	1.86	2.41	+29.52

^†^ The most and least deprived census blocks correspond to the third and first tertiles of the deprivation index distribution, respectively; ^‡^ The highest and lowest NO_2_ census blocks correspond to the third and first tertiles of the NO_2_ concentrations distribution, respectively; * For the three metropolitan areas and Paris; ** Calculated as: ((mortality at P2‰ − mortality at P1‰)/mortality at P1‰) × 100. Example: −15.5% = ((4.79‰ − 5.67‰)/5.67‰) × 100. --- There are no cases in Marseille during the first period, so the difference was not calculated.

**Table 3:** Summary of the spatial variation in infant mortality models in the Lille, Lyon and Marseille MAs and the city of Paris.

Infant Mortality		Lille				Lyon				Paris City				Marseille			
		P1 *		P2		P1		P2		P1		P2		P1		P2	
Unadjusted models	Test homogeneity global *p*-value **	0.001		0.003		0.155		0.001		0.018		0.019		0.001		0.013	
	Number of Significant Areas	2		1		0		1		1		1		2		1	
Adjusted models		Span	*p*-value	Span	*p*-value	Span	*p*-value	Span	*p*-value	Span	*p*-value	Span	*p*-value	Span	*p*-value	Span	*p*-value
Deprivation index		0.95	0.519 ^†^	0.85	0.173	0.95	0.368	0.95	0.024	0.95	0.394	0.95	0.163	0.75	0.004	0.85	0.103
NO_2_ concentrations		0.45	0.001	0.80	0.036	0.55	0.128	0.90	0.004	0.95	0.027	0.95	0.004	0.75	0.006	0.85	0.045
Deprivation index & NO_2_ concentrations		0.95	0.522	0.85	0.223	0.95	0.545	0.95	0.109	0.95	0.329	0.95	0.079	0.75	0.032	0.95	0.073

* For the three metropolitan areas and Paris, P1: 2002–2005, P2: 2006–2009; ** The global *p*-value denotes whether the smooth term for location is significant in the model. H_0_ means that there is no spatial variation of the estimated mortality risk. ^†^ A *p*-value > 0.05 means that a significant part of the spatial variability is explained by this covariate.

**Table 4:** Summary of the spatial variation in neonatal mortality models in the Lille, Lyon and Marseille MAs and the city of Paris.

Neonatal Mortality		Lille				Lyon				Paris City				Marseille			
		P1 *		P2		P1		P2		P1		P2		P1		P2	
**Unadjusted models**	Test homogeneity global *p*-value **	0.035		0.357		0.357		0.028		0.062		0.747		0.001		0.093	
	Number of Significant Areas	1		0		0		1		0		0		3		0	
**Adjusted models**		Span	*p*-value	Span	*p*-value	Span	*p*-value	Span	*p*-value	Span	*p*-value	Span	*p*-value	Span	*p*-value	Span	*p*-value
Deprivation index		0.95	**0.864** ^†^	0.95	0.679	0.95	0.494	0.95	**0.126**	0.95	0.466	0.95	0.948	0.75	0.002	0.95	0.422
NO_2_ concentrations		0.40	**0.173**	0.95	0.738	0.95	0.348	0.95	**0.059**	0.95	0.077	0.95	0.777	0.75	0.001	0.95	0.314
Deprivation index & NO_2_ concentrations		0.95	**0.911**	0.95	0.788	0.95	0.831	0.95	**0.304**	0.95	0.622	0.95	0.953	0.75	0.006	0.95	0.353

* For the three metropolitan areas and Paris, P1: 2002–2005, P2: 2006–2009; ** The global *p*-value denotes whether the smooth term for location is significant in the model. H_0_ means that there is no spatial variation of the estimated mortality risk; ^†^ A *p*-value > 0.05 means that a significant part of the spatial variability is explained by this covariate.
